# Sex Difference in the Associations among Obesity-Related Indices with Incident Hypertension in a Large Taiwanese Population Follow-Up Study

**DOI:** 10.3390/jpm12060972

**Published:** 2022-06-15

**Authors:** Wen-Chi Lee, Pei-Yu Wu, Jiun-Chi Huang, Yi-Chun Tsai, Yi-Wen Chiu, Szu-Chia Chen, Jer-Ming Chang, Hung-Chun Chen

**Affiliations:** 1 Department of General Medicine, Kaohsiung Medical University Hospital, Kaohsiung Medical University, Kaohsiung 807, Taiwan; wcsherry1211@gmail.com; 2 Division of Nephrology, Department of Internal Medicine, Kaohsiung Medical University Hospital, Kaohsiung Medical University, Kaohsiung 807, Taiwan; wpuw17@gmail.com (P.-Y.W.); karajan77@gmail.com (J.-C.H.); lidam65@yahoo.com.tw (Y.-C.T.); chiuyiwen@kmu.edu.tw (Y.-W.C.); jemich@kmu.edu.tw (J.-M.C.); chenhc@kmu.edu.tw (H.-C.C.); 3 Department of Internal Medicine, Kaohsiung Municipal Siaogang Hospital, Kaohsiung Medical University, Kaohsiung 812, Taiwan; 4 Faculty of Medicine, College of Medicine, Kaohsiung Medical University, Kaohsiung 807, Taiwan; 5 Research Center for Environmental Medicine, Kaohsiung Medical University, Kaohsiung 807, Taiwan

**Keywords:** obesity-related index, sex difference, incident hypertension, Taiwan Biobank, follow-up

## Abstract

Hypertension is a major risk factor for stroke, atherosclerosis, and other cardiovascular diseases, and obesity is a major risk factor for hypertension. The aim of this longitudinal study was to investigate sex differences in the correlations among obesity-related indices and incident hypertension in a large Taiwanese cohort. We included 21,466 enrollees in the Taiwan Biobank and followed them for 4 years. Of the 21,466 patients enrolled in this study, 6899 (mean age, 49.6 ± 10.9 years) were male and 14,567 (mean age, 49.7 ± 10.0 years) were female. Data on visceral adiposity index (VAI), waist-to-height ratio (WHtR), waist-to-hip ratio (WHR), lipid accumulation product (LAP), conicity index (CI), body roundness index (BRI), body mass index (BMI), body adiposity index (BAI), and abdominal volume index (AVI) were collected and analyzed. The results showed that all of the studied obesity-related indices were significantly associated with incident hypertension. Among them, WHtR was the strongest predictor of hypertension in both sexes. In addition, interactions between VAI, LAP, CI, BMI, and AVI with sex on incident hypertension were also statistically significant. CI and AVI were more strongly associated with hypertension in the men than in the women, while VAI, LAP, and BMI were more strongly associated with hypertension in the women. In conclusion, the studied obesity-related indices were found to be predictors of incident hypertension, and there were differences in the associations between the male and female participants. Our findings may imply that reducing body weight may be associated with a lower risk of developing hypertension.

## 1. Introduction

The prevalence of hypertension is increasing worldwide. A pooled analysis of 1201 studies with 104 million participants reported global hypertension prevalence rates of 32% in women and 34% in men aged 30–79 years [1]. In addition, the United States Centers for Disease Control (CDC) reported a hypertension prevalence rate of 45.4% in adults from 2017 to 2018, with a higher rate among men (51.0%) than women (39.7%), and that approximately 75% of adults aged 60 years and over had hypertension [2]. In Taiwan, the CDC reported that from 2013 to 2016 the prevalence rates of hypertension in adults were 28.38% among males and 21.96% among females, and that the prevalence was nearly 60% among patients aged over 60 years [3]. The pathophysiology of hypertension has yet to be fully elucidated, however proposed risk factors [4,5] include a high salt diet [6], insulin resistance [7], obesity [8,9,10], genetic factors [11], increased sympathetic nervous system (SNS) activity [12], renin-angiotensin system activation [13], and endothelial dysfunction [14]. Hypertension is a major cause of death in Taiwan, and a major risk factor for diseases including stroke [15], heart failure [16], renal failure [8], atherosclerosis [17], dementia [18], and further cardiovascular complications. Hence, the early detection and diagnosis of hypertension followed by early treatment is important to reduce cardiovascular risk, improve patient prognosis, and improve their quality of life.

In general, the diagnosis of hypertension is based on appropriately measured blood pressure (BP), followed by a complete clinical evaluation. Thus, there is a need for simple tools to quickly evaluate the presence of hypertension. Since obesity is a major risk factor for hypertension, it would be interesting to investigate the relationships between hypertension and obesity-related indices, as such indices can be easily calculated with laboratory findings and simple anthropometric measurements. In this study, we focused on visceral adiposity index (VAI), waist-to-height ratio (WHtR), waist-to-hip ratio (WHR), lipid accumulation product (LAP), conicity index (CI), body roundness index (BRI), body mass index (BMI), body adiposity index (BAI), and abdominal volume index (AVI), all of which can easily be calculated and quantified using measurements of high-density lipoprotein cholesterol (HDL-C), triglycerides (TGs), body weight (BW), body height (BH), hip circumference (HC), and waist circumference (WC). The calculation methods of obesity-related indices will be introduced at below. Among all, WHR and AVI are calculated by WC and HC, while WHtR, BRI, CI, and BAI use WC with BH. From the calculation methods, it seems that these indices take body shape into account, which may further reflect the degree of body fat centralization [19]. In addition, LAP is counted via WC and TG, with VAI calculated by WC, BMI, TG, and HDL. Both of them regard body shape and body fat component. As for BMI, it is calculated by body mass and height. 

These indices have been used as surrogate markers of insulin resistance and central obesity, and they have been shown to be highly correlated with metabolic syndrome and the risk of developing atherosclerotic cardiovascular diseases and diabetes mellitus (DM) [19,20,21]. Previous studies have also shown associations between these indices and albuminuria [22], lung function [23], osteoporosis [24], peripheral artery occlusive disease [25], poor cognitive function [26], and fatty liver [27]. Prior studies have also suggested that these obesity-related indices can be used as predictors of hypertension [28,29,30].

Few studies have evaluated sex differences in the relationships between these indices and incident hypertension. Therefore, the aim of this longitudinal study was to investigate sex differences in the correlations among obesity-related indices and incident hypertension in a large Taiwanese cohort.

## 2. Materials and Methods

### 2.1. Taiwan Biobank

The Ministry of Health and Welfare in Taiwan established the Taiwan Biobank (TWB) to promote healthcare, prevent chronic diseases, and address the increasingly aged population. The TWB includes medical, genetic, and lifestyle data on cancer-free adults aged 30 to 70 years residing in communities around Taiwan [31,32]. Ethical approval for the TWB was given by the Ethics and Governance Council of the TWB and Institutional Review Board on Biomedical Science Research, Academia Sinica, Taiwan.

When volunteers agree to be enrolled into the TWB, they undergo in-person interviews, physical examinations, and provide blood samples. During this enrollment process, data on WC, HC, BW, BH, BMI, age, sex, and personal medical histories (including hypertension and DM) are recorded, along with laboratory data of fasting glucose, hemoglobin, TGs, total cholesterol, HDL-C, LDL-C, estimated glomerular filtration rate (eGFR; calculated using the 4-variable Modification of Diet in Renal Disease study equation [33]), and uric acid.

In addition, BP is measured digitally by a trained member of staff, with each participant being asked to avoid caffeine, exercise, and smoking for at least 30 min before the first measurement. Each BP measurement is performed three times, with 1–2 min between each measurement, and the average systolic BP (SBP) and diastolic BP (DBP) values were used in the analysis in this study. Regular exercise was defined as participating in physical activity for at least 30 min at least three times per week. This study was conducted according to the Declaration of Helsinki, and approved by the Institutional Review Board of Kaohsiung Medical University Hospital (KMUHIRB-E(I)-20210058).

**Figure 1:**
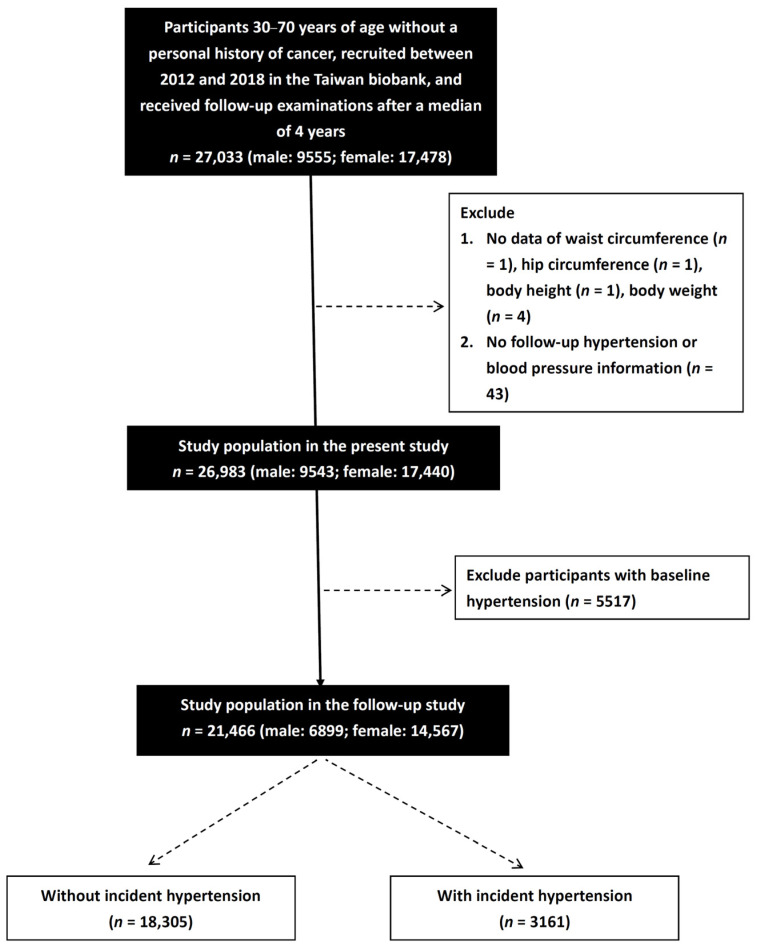
Flowchart of study population.

A total of 121,423 participants were included in the baseline study of TWB. We identified 27,033 participants (male: 9555; female: 17,478) in the TWB with follow-up data for a median of 4 years and excluded those with no data on WC (*n* = 1), HC (*n* = 1), BH (*n* = 1), and BW (*n* = 4), those with no follow-up data on hypertension or BP (*n* = 43), and those with baseline hypertension (*n* = 5517). The remaining 21,466 participants (male: 6899; female: 14,567) were enrolled (Figure 1), all of whom provided written informed consent.

### 2.2. Definition of Incident Hypertension

Participants with no past history of hypertension (self-reported) with a SBP < 140 mmHg and DBP < 90 mmHg were defined as not having hypertension. Those who developed hypertension (self-reported, SBP ≥ 140 mmHg, and DBP ≥ 90 mmHg) during follow-up were defined as having incident hypertension.

### 2.3. Calculation of Obesity-Related Indices

1. BMI was calculated as:

BMI = BW (kg)/BH^2^ (m)

2. WHR was calculated as:

WHR = WC (cm)/HC (cm)

3. WHtR was calculated as:

WHtR = WC (cm)/BH (cm)

4. BRI was calculated as:

BRI = $364.2-365.5\times \sqrt{1-{\left(\frac{\frac{{\mathrm{WC}}_{\left(m\right)}}{2\pi }}{0.5\times {\mathrm{BH}}_{\left(m\right)}}\right)}^{2}}$ [34].

5. CI was calculated using the Valdez equation based on BW, BH and WC as:

CI = $\frac{{\mathrm{WC}}_{\left(m\right)}}{0.109\times \sqrt{\frac{{\mathrm{BW}}_{\left(\mathrm{kg}\right)}}{{\mathrm{BH}}_{\left(m\right)}}}\;}\;$ [35].

6. BAI was calculated according to the method of Bergman and colleagues as:

BAI = $\frac{{\mathrm{HC}}_{\left(\mathrm{cm}\right)}}{{\mathrm{BH}}_{\left(m\right)}{}^{3/2}}-18$ [36].

7. AVI was calculated as AVI = $\frac{2\times {\left({\mathrm{WC}}_{\left(\mathrm{cm}\right)}\right)}^{2}+0.7\times {\left({\mathrm{WC}}_{\left(\mathrm{cm}\right)}-{\mathrm{HC}}_{\left(\mathrm{cm}\right)}\right)}^{2}}{1000}$ [37].

8. LAP was calculated as:

LAP = $\left({\mathrm{WC}}_{\left(\mathrm{cm}\right)}-65\right)\times {\mathrm{TG}}_{\left(\mathrm{mmol}/L\right)}$ in males, and

LAP = $\left({\mathrm{WC}}_{\left(\mathrm{cm}\right)}-58\right)\times {\mathrm{TG}}_{\left(\mathrm{mmol}/L\right)}$ in females [38].

9. VAI score was calculated as described previously [39] using the following sex-specific equations (with TG levels in mmol/L and HDL-cholesterol levels in mmol/L):

VAI = $\left(\frac{{\mathrm{WC}}_{\left(\mathrm{cm}\right)}}{39.68+\left(1.88\times \mathrm{BMI}\right)}\right)\times \left(\frac{{\mathrm{TG}}_{\left(\mathrm{mmol}/L\right)}}{1.03}\right)\times \left(\frac{1.31}{{\mathrm{HDL}}_{\left(\mathrm{mmol}/L\right)}}\right)$ in males, and

VAI = $\left(\frac{{\mathrm{WC}}_{\left(\mathrm{cm}\right)}}{36.58+\left(1.89\times \;\mathrm{BMI}\right)}\right)\times \left(\frac{{\mathrm{TG}}_{\left(\mathrm{mmol}/L\right)}}{0.81}\right)\times \left(\frac{1.52}{{\mathrm{HDL}}_{\left(\mathrm{mmol}/L\right)}}\right)$ in females.

### 2.4. Statistical Analysis

Statistical analysis was done using the Statistical Package for Social Sciences software package (version 19 for Windows^®^, IBM Inc., Armonk, NY, USA). Data are presented as percentage or mean ± standard deviation. Differences between continuous variables were compared using the independent *t*-test, and differences between categorical variables were compared using the Chi-square test. Univariable, age-adjusted, and multivariable logistic regression analysis was used to identify factors associated with incident hypertension. Significant variables in univariable analysis were put into multivariable analysis. Receiver operating characteristic (ROC) curves were plotted to assess the performance of the obesity-related indices to identify incident hypertension, and areas under the ROC curves (AUCs) were used to assess their predictive ability. A difference was considered significant if the *p* value was less than 0.05.

## 3. Results

Of the 21,466 patients (mean age, 49.7 ± 10.3 years) enrolled in this study, 6899 were male and 14,567 were female. The prevalence rates of incident hypertension were 19.8% and 12.3% in the males and females (*p* < 0.001), respectively.

### 3.1. Comparisons of Clinical Characteristics between the Participants by Sex

Table 1 showed the comparison of clinical characteristics between male and female participants. Compared to the male participants, female participants had low prevalence of DM, a smoking and alcohol consumption history, regular exercise habit, lower SBP, DBP, BH, BW, WC, HC, fasting glucose, hemoglobin, TGs, LDL-C, uric acid, BMI, WHR, BRI, CI, AVI, LAP, and VAI, and higher total cholesterol, HDL-C, eGFR, and BAI.

### 3.2. Comparisons of Clinical Characteristics between the Participants with and without Incident Hypertension by Sex

Compared to the male participants without incident hypertension, more of the male participants with incident hypertension were older, had DM, a history of smoking and alcohol consumption, and regular exercise habit (Table 2). In addition, the male participants with incident hypertension had higher SBP, DBP, LDL-C, uric acid, BW, WC, HC, fasting glucose, hemoglobin, TGs, and total cholesterol, and lower BH, HDL-C, and eGFR than those without incident hypertension (Table 2). Regarding the obesity-related indices, the male participants with incident hypertension also had higher VAI, WHtR, WHR, LAP, CI, BRI, BMI, BAI, and AVI. Similar results were found in the female participants, except there was no significant association between a history of alcohol consumption and incident hypertension.

### 3.3. Associations among Obesity-Related Indices with Incident Hypertension by Sex

Univariable and age-adjusted logistic regression analysis was used to identify associations among the obesity-related indices with incident hypertension by sex, and the results are shown in Table 3 (male) and Table 4 (female). All obesity-related indices are associated with incident hypertension, regardless of univariable and age-adjusted models in both sexes.

Multivariable logistic regression analysis was used to identify associations among the obesity-related indices with incident hypertension by sex, and the results are shown in Table 5. The following models were used:

1. For WHtR, WHR, CI, BRI, BMI, BAI, and AVI: adjustments for age, DM, smoking and alcohol history, regular exercise habit, fasting glucose, hemoglobin, TGs, total cholesterol, HDL-C, LDL-C, eGFR, and uric acid.

2. For LAP: adjustments for age, DM, smoking and alcohol history, regular exercise habit, fasting glucose, hemoglobin, total cholesterol, HDL-C, LDL-C, eGFR, and uric acid.

3. For VAI: adjustments for age, DM, smoking and alcohol history, regular exercise habit, fasting glucose, hemoglobin, total cholesterol, LDL-C, eGFR, and uric acid.

The results of the analyses showed that high values of VAI (per 1; odds ratio [OR] = 1.084), WHtR (per 0.01; OR = 1.062), WHR (per 0.01; OR = 1.038), LAP (per 1; OR = 1.008), CI (per 0.1; OR = 1.237), BRI (per 1; OR = 1.190), BMI (per 1 kg/m^2^; OR = 1.107), BAI (per 1; OR = 1.084), and AVI (per 1; OR = 1.092) were significantly associated with incident hypertension in the male participants (all *p* < 0.001). Similarly, high values of VAI (per 1; OR = 1.095), WHtR (per 0.01; OR = 1.053), WHR (per 0.01; OR = 1.027), LAP (per 1; OR = 1.010), CI (per 0.1; OR = 1.095), BRI (per 1; OR = 1.166), BMI (per 1 kg/m^2^; OR = 1.119), BAI (per 1; OR = 1.078), and AVI (per 1; OR = 1.090) were also significantly associated with incident hypertension in the female participants (all *p* < 0.001 except for CI (*p* = 0.005)).

### 3.4. Interactions among Obesity-Related Indices and Sex on Incident Hypertension

There were significant interactions between sex and BMI (*p* = 0.010), CI (*p* = 0.008), AVI (*p* = 0.013), LAP (*p* < 0.001), and VAI (*p* = 0.002) on incident hypertension (Table 2). However, no significant differences were found in the other indices.

We have further tried to analyze the two separated models. One was unadjusted factors, such as DM, fasting glucose, TG, total cholesterol, HDL-C, and LDL-C; another one was the multivariable adjusted model. We found that there was no overlap in the 95% confidence intervals of each obesity-related indices between the two models (Supplemental Tables S1 and S2).

### 3.5. Performance and Predictive Ability of the Obesity-Related Indices to Identify Incident Hypertension by Sex

**Figure 2:**
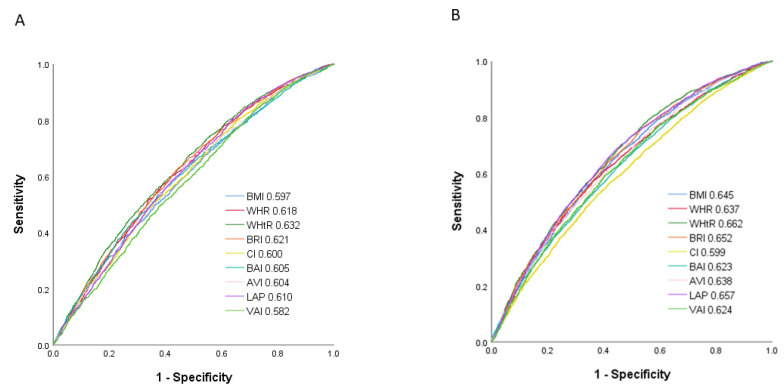
Comparison of the predictive value of nine obesity-related indices for diagnosis of incident hypertension among (**A**) males and (**B**) females.

The performance (ROC curves) and predictive ability (AUCs) of the nine obesity-related indices to identify incident hypertension by sex are shown in Figure 2. In the male participants, WHtR had the highest AUC (0.632), followed by BRI (0.621), WHR (0.618), LAP (0.610), BAI (0.605), AVI (0.604), CI (0.600), BMI (0.597) and VAI (0.582). In the female participants, WHtR also had the highest AUC (0.662), followed by LAP (0.657), BRI (0.652), BMI (0.645), AVI (0.638), WHR (0.637), VAI (0.624), and CI (0.599).

The AUCs, cutoff values, Youden index, sensitivity, and specificity of the obesity-related indices to identify incident hypertension in the male and female participants are shown in Table 6 and Table 7, respectively.

## 4. Discussion

In this longitudinal analysis, we investigated sex differences in the relationships among obesity-related indices with incident hypertension in 21,466 Taiwanese participants with a median 4 years of follow-up. The results showed that all of the studied obesity-related indices were significantly associated with incident hypertension in both the male and female participants. WHtR had the highest AUC for the prediction of incident hypertension, followed by BRI and WHR in the male participants, compared to WHtR, LAP, and BRI in the female participants. In addition, the interactions between VAI, LAP, CI, BMI, and AVI with sex on incident hypertension were also statistically significant. CI and AVI were more strongly associated with incident hypertension in the men than in the women, while BMI, LAP, and VAI were more strongly associated with incident hypertension in the women.

There are several important findings of this study. First, high obesity-related indices were significantly associated with incident hypertension in both the male and female participants. The results were consistent with other studies [28,29,30]. In the Johns Hopkins Precursors Study, 1132 white men, with median follow-up period of 46 years, demonstrated strong evidence for the association between BMI and incident hypertension [40]. Chua et al. collected data from the villages of Peninsular Malaysia, revealing that WC, WHtR, and WHR are similarly useful as predictors of hypertension in men and women [41]. A study conducted among Singaporean supported that WC and WHtR had equal power in predicting hypertension, irrespective of age, sex, and ethnicity [42]. Hence, obesity appears to be an important risk factor for hypertension, and similar results could be seen [10]. The Framingham Offspring Study confirmed that body fat changes over an 8-year period were significantly related to both SBP and DBP changes in both sexes [43]. The beneficial effects of weight loss on BP have also been demonstrated in other studies, regardless of the weight loss strategy (lifestyle modification, diet control, pharmacological intervention, or bariatric surgery) [44]. Several mechanisms may explain the association between obesity and hypertension, including a physical compression effect of visceral and retroperitoneal fat on the kidneys, increased SNS activity, activation of the renin-angiotensin-aldosterone system (RAAS), and also metabolic disorders (including insulin resistance, dyslipidemia, and inflammation) [8,10]. With regards to renal compression, excess perirenal fat and renal sinus fat may compress the kidneys and thereby increase intrarenal pressure. This could then cause reduced blood flow in the kidney medulla contributing to increased sodium reabsorption, renin secretion, and glomerular hyperfiltration [8,10]. In addition, excessive fat may cause lipotoxicity to some degree [45]. On the other hand, several studies have suggested associations between obesity and increased angiotensin II and aldosterone levels, which have in turn been associated with kidney compression and SNS activation [8,10]. Another study also indicated that adipose tissue may play a role in RAAS activation [46]. In addition, obesity can cause an increase in leptin, which can then increase SNS activity. Obesity can also cause obstructive sleep apnea and subsequently hypoxemia, hypercapnia, and chemoreceptor activation, which would further stimulate SNS activity [8,10]. With regard to the pathophysiology of metabolic syndrome-related hypertension, insulin resistance may lead to endothelial dysfunction, induce inflammation in soft tissues, and impair the production of nitric oxide [47]. Conversely, endothelial dysfunction could cause insulin resistance, and thus worsen endothelial function in a vicious circle [48,49]. Insulin can also influence SNS and renal function [48]. In summary, the proposed mechanisms would increase renal sodium reabsorption and cause sodium retention, followed by an expansion of extracellular fluid volume, ultimately resulting in elevated BP. An elevated BP would then cause further renal injury and cardiovascular disease, which in turn would gradually increase BP further causing hypertension to be more resistant to treatment. Although we tried to use insulin resistance to partly explain the association of obesity index with hypertension, in this study, we lacked the data of insulin resistance, such as Homeostatic Model Assessment for Insulin Resistance. Therefore, the pathophysiology of obesity-induced hypertension is complex and has yet to be fully elucidated, and further studies are needed to clarify this issue.

In our study, many factors as confounding factors were enrolled, and the risk of multi-covariance could be elevated. From our crude and age-adjusted model (Table 3; Table 4) and unadjusted insulin resistance related factors (supplementary Tables S1 and S2), we still found that all obesity-related indices were significantly associated with incident hypertension. There was no overlap in the 95% confidence intervals of each obesity-related indices between these models, which may imply that the risk of multi-covariance was not elevated. The influence of multi-covariance in the present model could be decreased.

As obesity is significantly associated with hypertension, it would be valuable to identify anthropometric obesity-related indices which could be used to evaluate and diagnose hypertension at an early stage. Among the obesity-related indices included in this study, we found that WHtR had the highest AUC for the prediction of incident hypertension, followed by BRI and WHR in the male participants, compared to WHtR, LAP, and BRI in the female participants. In both sexes, WHtR had the strongest predictive ability for incident hypertension, which is consistent with previous studies [19,20,21,29,50]. In a prospective cohort study of Korean adults, the WHtR cutoff value for predicting DM, cardiovascular disease, hypertension, and metabolic syndrome was 0.5 [21]. In another study of Japanese community-dwelling middle-aged and elderly individuals, WHtR cutoff values of 0.47 for men and 0.53 for women were strongly associated with hypertension [19]. These values are similar to our findings, in which a WHtR cutoff value of 0.51 was a good predictor of incident hypertension in both sexes. A possible reason why WHtR, BRI, WHR, and LAP were stronger predictors than BMI may be because these indices are markers that take differences in body distribution of adiposity into consideration, and thus reflect the degree of body fat centralization [19]. In contrast, BMI is used as an index of general body mass and may not be suitable to evaluate obesity-associated diseases [51], as it tends to reveal weight overaccumulation, but cannot fully reflect the component ratio of fat and muscle.

Another important finding of this study is that interactions between VAI, LAP, CI, BMI, and AVI with sex on incident hypertension were statistically significant. Moreover, CI and AVI were more strongly associated with incident hypertension in the men than in the women, while BMI, LAP, and VAI were more strongly associated with incident hypertension in the women. Sex differences in obesity indices related to the risk of hypertension have been reported in previous studies [52,53]. However, there is currently no consensus on which obesity-related index is the strongest predictor in either sex. The underlying mechanisms are probably complex. One possible mechanism may be that females have higher leptin and leptin receptor expressions than males. These factors play a role in fat distribution and result in more predominant subcutaneous adipose fat than visceral fat in females, which may result in a higher BMI in females than in males [52,54]. Another possible explanation may be that females tend to accumulate fat on their thighs and buttocks, whereas males are more prone to abdominal fat deposits [55]. A study enrolled 29,079 participants in China supported similar view, demonstrating that visceral obesity tends to be more significant in men with regard to hypertension, while the better indicator for hypertension in women should be overall obesity [56]. These sex differences in fat distribution may correspond with WC, and thus result in relatively higher WHR, CI, and AVI in men than in women. Similar issues have been reported in Nigeria, revealing that the anthropometric indices of central adiposity (WHR, CI, AVI, WC, WHtR) may be more predictive of hypertension [57]. In addition, other factors associated with sex differences include diet, behavioral factors, ethnicity, environmental factors, sex hormones, and sex-specific gene expressions [58].

In our present study, the participants were recruited 30–70 years of age, during 2012 and 2018, with follow-up after a median of 4 years. The participants with baseline hypertension were 5517 among 26,983 people. Among all, the prevalence rate of hypertension in male participants was 27.7% (2644 in 9543), and 16.5% (2873 in 17,440) in female participants. The age was mildly lower than the report which collected participants above 18 years old during 2013 to 2016 by Taiwan Health Promotion Administration, revealing 28.38% for men and 21.96% for women. One possible explanation is that the median age in our study is 49.6 ± 10.9 in male, and 49.7 ± 10.0 in female, while the study conducted by Taiwan Health Promotion Administration located the median age in the 45 to 64 group [3,59]. The representativeness of the present study population needs to be noted.

The main strengths of this study are that we included a large population and addressed sex differences in the associations among nine obesity-related indices and incident hypertension. Despite these strengths, there are also several limitations. First, we could not analyze the effects of medications including lipid-lowering agents or anti-hypertensive and anti-diabetic agents, as such data are not provided by the TWB. These drugs may have had an effect on incident hypertension, and therefore the associations identified in this study may be underestimated. Second, we also lacked data on proteinuria, which has been associated with incident hypertension. Third, the TWB only includes information on participants of Chinese ethnicity, and thus our findings should be applied with caution to other ethnicities. Finally, as only approximately 25% of participants in the TWB undergo follow-up evaluations, sample bias may be an issue.

In conclusion, we found significant associations among the nine studied obesity-related indices (VAI, WHtR, WHR, LAP, CI, BRI, BMI, BAI, and AVI) and incident hypertension in both male and female participants. Among them, WHtR was the strongest predictor of hypertension in both sexes. Furthermore, the interactions between VAI, LAP, CI, BMI, and AVI with sex on incident hypertension were also statistically significant. CI and AVI were more strongly associated with hypertension in the men than in the women, while BMI, LAP, and VAI were more strongly associated with hypertension in the women. Our results showed that the studied obesity-related indices were predictors of incident hypertension, and there were differences in the associations between the male and female participants. Our findings may imply that reducing body weight may be associated with a lower risk of developing hypertension.

## Figures and Tables

**Table 1:** Clinical characteristics of the study participants classified by sex.

Characteristics	Male (*n* = 6899)	Female (*n* = 14,567)	*p*
Age (year)	49.6 ± 10.9	49.7 ± 10.0	0.465
DM (%)	5.0	2.8	<0.001
Smoking history (%)	57.8	7.9	<0.001
Alcohol history (%)	6.9	0.7	<0.001
Regular exercise habits (%)	46.2	46.1	0.876
Systolic BP (mmHg)	116.1 ± 11.5	110.0 ± 13.3	<0.001
Diastolic BP (mmHg)	73.8 ± 8.2	67.9 ± 8.7	<0.001
Body height (cm)	169.2 ± 6.3	157.2 ± 5.5	<0.001
Body weight (Kg)	70.6 ± 10.6	57.3 ± 8.9	<0.001
Waist circumference (cm)	86.1 ± 8.5	79.9 ± 9.1	<0.001
Hip circumference (cm)	96.6 ± 6.2	94.6 ± 6.6	<0.001
Laboratory parameters			
Fasting glucose (mg/dL)	98.2 ± 22.2	92.8 ± 16.5	<0.001
Hemoglobin (g/dL)	15.0 ± 1.1	13.0 ± 1.3	<0.001
Triglyceride (mg/dL)	128.6 ± 98.9	98.1 ± 67.0	<0.001
Total cholesterol (mg/dL)	191.8 ± 34.4	196.6 ± 35.7	<0.001
HDL-cholesterol (mg/dL)	48.6 ± 11.1	58.4 ± 13.0	<0.001
LDL-cholesterol (mg/dL)	122.7 ± 31.4	120.7 ± 31.7	<0.001
eGFR (mL/min/1.73 m^2^)	100.9 ± 20.0	116.0 ± 25.6	<0.001
Uric acid (mg/dL)	6.4 ± 1.3	4.8 ± 1.1	<0.001
Obesity-related indices			
BMI (kg/m^2^)	24.6 ± 3.2	23.2 ± 3.4	<0.001
WHR (%)	89.0 ± 5.4	84.3 ± 6.6	<0.001
WHtR (%)	50.9 ± 5.0	50.8 ± 6.0	0.335
BRI	6.9 ± 1.6	6.3 ± 1.8	<0.001
CI	1.22 ± 0.06	1.22 ± 0.08	<0.001
BAI	26.0 ± 3.0	30.1 ± 3.7	<0.001
AVI	15.1 ± 3.0	13.1 ± 2.9	<0.001
LAP	33.1 ± 33.7	26.1 ± 25.4	<0.001
VAI	1.67 ± 1.75	1.55 ± 1.52	<0.001

Abbreviations: DM, diabetes mellitus; BP, blood pressure; HDL, high-density lipoprotein; LDL, low-density lipoprotein; eGFR, estimated glomerular filtration rate; BMI, body mass index; WHR, waist–hip ratio; WHtR, waist-to-height ratio; BRI, body roundness index; CI, conicity index; BAI, body adiposity index; AVI, abdominal volume index; LAP, lipid accumulation product; VAI, visceral adiposity index.

**Table 2:** Clinical characteristics of the study participants classified by the presence of different sex and incident hypertension.

Characteristics		Male (*n* = 6899)		Female (*n* = 14,567)		
	Incident Hypertension (−) (*n* = 5530)	Incident Hypertension (+) (*n* = 1369)	*p*	Incident Hypertension (−) (*n* = 12,775)	Incident Hypertension (+) (*n* = 1792)	*p*
Age (year)	48.5 ± 10.9	54.0 ± 10.0	<0.001	48.9 ± 9.9	55.5 ± 8.5	<0.001
DM (%)	4.3	7.7	<0.001	2.5	5.7	<0.001
Smoking history (%)	56.9	61.7	0.001	8.2	5.7	<0.001
Alcohol history (%)	6.5	8.9	0.001	0.7	0.6	0.615
Regular exercise habits (%)	44.8	52.0	<0.001	45.1	53.3	<0.001
Systolic BP (mmHg)	113.9 ± 10.9	124.8 ± 9.4	<0.001	108.1 ± 12.4	124.0 ± 10.5	<0.001
Diastolic BP (mmHg)	72.7 ± 8.0	78.5 ± 7.4	<0.001	66.9 ± 8.3	74.9 ± 8.3	<0.001
Body height (cm)	169.5 ± 6.2	167.9 ± 6.4	<0.001	157.5 ± 5.6	155.7 ± 5.2	<0.001
Body weight (Kg)	70.2 ± 10.5	72.0 ± 10.7	<0.001	56.9 ± 8.7	59.8 ± 9.7	<0.001
Waist circumference (cm)	85.5 ± 8.5	88.5 ± 8.2	<0.001	79.3 ± 8.9	83.7 ± 9.4	<0.001
Hip circumference (cm)	96.4 ± 6.2	97.5 ± 6.3	<0.001	94.4 ± 6.5	96.1 ± 7.3	<0.001
Laboratory parameters						
Fasting glucose (mg/dL)	97.3 ± 20.6	101.7 ± 27.4	<0.001	92.1 ± 15.4	97.8 ± 22.7	<0.001
Hemoglobin (g/dL)	15.0 ± 1.1	15.1 ± 1.2	0.038	12.9 ± 1.3	13.2 ± 1.3	<0.001
Triglyceride (mg/dL)	124.7 ± 97.0	144.3 ± 104.7	<0.001	95.4 ± 62.4	117.9 ± 90.7	<0.001
Total cholesterol (mg/dL)	191.1 ± 34.5	194.9 ± 33.8	<0.001	195.7 ± 35.5	203.0 ± 36.5	<0.001
HDL-cholesterol (mg/dL)	49.0 ± 11.3	47.2 ± 10.5	<0.001	58.7 ± 13.0	55.8 ± 12.7	<0.001
LDL-cholesterol (mg/dL)	122.2 ± 31.3	124.7 ± 31.9	<0.001	119.9 ± 31.4	126.6 ± 33.1	<0.001
eGFR (mL/min/1.73 m^2^)	101.8 ± 19.7	97.4 ± 21.1	<0.001	116.8 ± 25.5	110.2 ± 25.6	<0.001
Uric acid (mg/dL)	6.3 ± 1.3	6.6 ± 1.4	<0.001	4.8 ± 1.0	5.2 ± 1.1	<0.001
Obesity-related indices						
BMI (kg/m^2^)	24.4 ± 3.1	25.5 ± 3.1	<0.001	22.9 ± 3.3	24.6 ± 3.6	<0.001
WHR (%)	88.6 ± 5.4	90.7 ± 5.0	<0.001	84.0 ± 6.5	87.1 ± 6.5	<0.001
WHtR (%)	50.5 ± 5.0	52.7 ± 4.8	<0.001	50.4 ± 5.9	53.8 ± 6.1	<0.001
BRI	6.8 ± 1.6	7.4 ± 1.6	<0.001	6.2 ± 1.7	7.1 ± 1.9	<0.001
CI	1.22 ± 0.06	1.24 ± 0.06	<0.001	1.21 ± 0.08	1.24 ± 0.09	<0.001
BAI	25.8 ± 2.9	26.8 ± 3.1	<0.001	29.9 ± 3.6	31.5 ± 3.9	<0.001
AVI	14.9 ± 2.9	15.9 ± 2.9	<0.001	12.9 ± 2.9	14.3 ± 3.2	<0.001
LAP	31.3 ± 32.3	40.4 ± 37.9	<0.001	24.7 ± 23.3	35.8 ± 35.7	<0.001
VAI	1.61 ± 1.72	1.93 ± 1.86	<0.001	1.49 ± 1.40	1.97 ± 2.19	<0.001

Abbreviations: DM, diabetes mellitus; BP, blood pressure; HDL, high-density lipoprotein; LDL, low-density lipoprotein; eGFR, estimated glomerular filtration rate; BMI, body mass index; WHR, waist–hip ratio; WHtR, waist-to-height ratio; BRI, body roundness index; CI, conicity index; BAI, body adiposity index; AVI, abdominal volume index; LAP, lipid accumulation product; VAI, visceral adiposity index.

**Table 3:** Association of obesity-related indices with incident hypertension using univariable and age-adjusted logistic regression analysis in male participants.

Obesity-Related Indices	Male (*n* = 6899)			Male (*n* = 6899)		
	Crude			Age-Adjusted		
	OR	95% Confidence Interval	*p*	OR	95% Confidence Interval	*p*
BMI (per 1 kg/m^2^)	1.108	1.088–1.128	<0.001	1.140	1.118–1.162	<0.001
WHR (per 0.01)	1.077	1.065–1.090	<0.001	1.060	1.048–1.072	<0.001
WHtR (per 0.01)	1.092	1.079–1.105	<0.001	1.083	1.070–1.097	<0.001
BRI (per 1)	1.267	1.222–1.313	<0.001	1.265	1.219–1.313	<0.001
CI (per 0.1)	1.722	1.565–1.894	<0.001	1.455	1.318–1.606	<0.001
BAI (per 1)	1.127	1.105–1.150	<0.001	1.113	1.091–1.136	<0.001
AVI (per 1)	1.116	1.095–1.138	<0.001	1.130	1.108–1.154	<0.001
LAP (per 1)	1.007	1.005–1.009	<0.001	1.008	1.007–1.010	<0.001
VAI (per 1)	1.092	1.058–1.127	<0.001	1.111	1.074–1.149	<0.001

Values expressed as odds ratio (OR) and 95% confidence interval. Abbreviations are the same as in Table 1.

**Table 4:** Association of obesity-related indices with incident hypertension using univariable and age-adjusted logistic regression analysis in female participants.

Obesity-Related Indices	Female (*n* = 14,567)			Female (*n* = 14,567)		
	Crude			Age-Adjusted		
	OR	95% Confidence Interval	*p*	OR	95% Confidence Interval	*p*
BMI (per 1 kg/m^2^)	1.141	1.126–1.157	<0.001	1.150	1.134–1.167	<0.001
WHR (per 0.01)	1.072	1.064–1.080	<0.001	1.044	1.036–1.052	<0.001
WHtR (per 0.01)	1.091	1.082–1.099	<0.001	1.071	1.062–1.080	<0.001
BRI (per 1)	1.293	1.261–1.325	<0.001	1.235	1.203–1.269	<0.001
CI (per 0.1)	1.483	1.401–1.570	<0.001	1.204	1.134–1.278	<0.001
BAI (per 1)	1.119	1.105–1.134	<0.001	1.104	1.089–1.119	<0.001
AVI (per 1)	1.151	1.134–1.169	<0.001	1.130	1.112–1.149	<0.001
LAP (per 1)	1.014	1.012–1.015	<0.001	1.011	1.009–1.012	<0.001
VAI (per 1)	1.168	1.134–1.203	<0.001	1.123	1.091–1.157	<0.001

Values expressed as odds ratio (OR) and 95% confidence interval. Abbreviations are the same as in Table 1.

**Table 5:** Association of obesity-related indices with incident hypertension using multivariable logistic regression analysis.

Obesity-Related Indices	Male (*n* = 6899)			Female (*n* = 14,567)			
	Multivariable			Multivariable			
	OR	95% Confidence Interval	*p*	OR	95% Confidence Interval	*p*	Interaction *p*
BMI (per 1 kg/m^2^) ^a^	1.107	1.083–1.131	<0.001	1.119	1.101–1.137	<0.001	0.010
WHR (per 0.01) ^a^	1.038	1.024–1.051	<0.001	1.027	1.018–1.035	<0.001	0.477
WHtR (per 0.01) ^a^	1.062	1.048–1.077	<0.001	1.053	1.043–1.062	<0.001	0.890
BRI (per 1) ^a^	1.190	1.141–1.241	<0.001	1.166	1.131–1.201	<0.001	0.369
CI (per 0.1) ^a^	1.237	1.112–1.375	<0.001	1.095	1.028–1.166	0.005	0.008
BAI (per 1) ^a^	1.084	1.061–1.107	<0.001	1.078	1.062–1.093	<0.001	0.574
AVI (per 1) ^a^	1.092	1.068–1.117	<0.001	1.090	1.071–1.110	<0.001	0.013
LAP (per 1) ^b^	1.008	1.005–1.111	<0.001	1.010	1.007–1.013	<0.001	<0.001
VAI (per 1) ^c^	1.084	1.041–1.128	<0.001	1.095	1.062–1.128	<0.001	0.002

Values expressed as odds ratio (OR) and 95% confidence interval. Abbreviations are the same as in Table 1. ^a^ Covariates in the multivariable model included age, diabetes, smoking and alcohol history, regular exercise habits, fasting glucose, hemoglobin, triglyceride, total cholesterol, HDL-cholesterol, LDL-cholesterol, eGFR, and uric acid. ^b^ Covariates in the multivariable model included age, diabetes, smoking and alcohol history, regular exercise habits, fasting glucose, hemoglobin, total cholesterol, HDL-cholesterol, LDL-cholesterol, eGFR, and uric acid. ^c^ Covariates in the multivariable model included age, diabetes, smoking and alcohol history, regular exercise habits, fasting glucose, hemoglobin, total cholesterol, LDL-cholesterol, eGFR, and uric acid.

**Table 6:** Area under curve (AUC), cutoff value, sensitivity, specificity, and Youden index of nine obesity-related indices in male participants.

Obesity-Related Indices	AUC (95% Confidence Interval)	Cutoff Value	Sensitivity (%)	Specificity (%)	Youden Index
BMI (kg/m^2^)	0.597 (0.581–0.614) *	24.700	56.7	56.7	0.134
WHR	0.618 (0.602–0.634) *	0.897	58.7	58.7	0.174
WHtR	0.632 (0.616–0.648) *	0.514	59.3	59.1	0.184
BRI	0.621 (0.605–0.637) *	6.952	58.7	58.8	0.175
CI	0.600 (0.584–0.617) *	1.232	57.0	57.0	0.140
BAI	0.605 (0.588–0.621) *	26.166	57.9	57.8	0.157
AVI	0.604 (0.588–0.620) *	15.138	58.1	58.1	0.162
LAP	0.610 (0.594–0.626) *	26.802	58.1	58.1	0.162
VAI	0.582 (0.566–0.599) *	1.287	55.6	55.6	0.112

* *p* < 0.001. Abbreviations are the same as in Table 1.

**Table 7:** Area under curve (AUC), cutoff value, sensitivity, specificity, and Youden index of nine obesity-related indices in female participants.

Obesity-Related Indices	AUC (95% Confidence Interval)	Cutoff Value	Sensitivity (%)	Specificity (%)	Youden Index
BMI (kg/m^2^)	0.645 (0.632–0.658) *	23.318	60.7	60.7	0.214
WHR	0.637 (0.624–0.651) *	0.852	60.4	60.3	0.207
WHtR	0.662 (0.649–0.674) *	0.516	61.5	61.5	0.230
BRI	0.652 (0.639–0.666) *	6.409	60.9	60.8	0.217
CI	0.599 (0.585–0.613) *	1.222	57.2	57.2	0.144
BAI	0.623 (0.610–0.637) *	30.266	58.4	58.4	0.168
AVI	0.638 (0.624–0.651) *	13.151	59.6	59.6	0.192
LAP	0.657 (0.644–0.670) *	23.080	61.7	61.7	0.234
VAI	0.624 (0.610–0.637) *	1.304	59.4	59.4	0.188

* *p* < 0.001. Abbreviations are the same as in Table 1.

## Data Availability

The data underlying this study are from the Taiwan Biobank. Due to restrictions placed on the data by the Personal Information Protection Act of Taiwan, the minimal data set cannot be made publicly available. Data may be available upon request to interested researchers. Please send data requests to: Szu-Chia Chen, PhD, MD. Division of Nephrology, Department of Internal Medicine, Kaohsiung Medical University Hospital, Kaohsiung Medical University.

## Supplementary Materials

The following supporting information can be downloaded at: https://www.mdpi.com/article/10.3390/jpm12060972/s1, Table S1: Comparison of the association of obesity-related indices with incident hypertension using different logistic regression analysis in male participants; Table S2: Comparison of the association of obesity-related indices with incident hypertension using different logistic regression analysis in female participants.
