# Supplementary material for: Sex Difference in the Associations among Obesity-Related Indices with Incident Hypertension in a Large Taiwanese Population Follow-Up Study
Source: J Pers Med. 2022 Jun 15;12(6):972. doi: 10.3390/jpm12060972 (PMC9225143; doi:10.3390/jpm12060972)
Supplement: Supplementary file 1 [file jpm-12-00972-s001.zip › jpm-1767127-supplementary.pdf]

Supplemental Table S1. Comparison of the association of obesity-related indices with incident hypertension using different logistic regression analysis in male participants

Obesity-related indices	Male (n = 6899)			Male (n = 6899)		
	Multivariable[#]			Multivariable[a,b,c]		
	OR	95% confidence interval	p	OR	95% confidence interval	p
BMI (per 1 kg/m^2)	1.118[#]	1.095-1.141	< 0.001	1.107[a]	1.083-1.131	< 0.001
WHR (per 0.01)	1.047[#]	1.034-1.060	< 0.001	1.038[a]	1.024-1.051	< 0.001
WHtR (per 0.01)	1.070[#]	1.056-1.084	< 0.001	1.062[a]	1.048-1.077	< 0.001
BRI (per 1)	1.217[#]	1.170-1.266	< 0.001	1.190[a]	1.141-1.241	< 0.001
CI (per 0.1)	1.324[#]	1.195-1.467	< 0.001	1.237[a]	1.112-1.375	< 0.001
BAI (per 1)	1.094[#]	1.072-1.117	< 0.001	1.084[a]	1.061-1.107	< 0.001
AVI (per 1)	1.106[#]	1.082-1.130	< 0.001	1.092[a]	1.068-1.117	< 0.001
LAP (per 1)	1.006[#]	1.004-1.008	< 0.001	1.008[b]	1.005-1.111	< 0.001
VAI (per 1)	1.069[#]	1.034-1.104	< 0.001	1.084[c]	1.041-1.128	< 0.001

Values expressed as odds ratio (OR) and 95% confidence interval. Abbreviations are the same as in Table 1.

[#]Covariates in the multivariable model included age, smoking and alcohol history, regular exercise habits, hemoglobin, eGFR and uric acid for BMI, WHR, WHtR, BRI, CI. BAI, AVI, LAP and VAI.

[a]Covariates in the multivariable model included age, diabetes, smoking and alcohol history, regular exercise habits, fasting glucose, hemoglobin,

triglyceride, total cholesterol, HDL-cholesterol, LDL-cholesterol, eGFR and uric acid for BMI, WHR, WHtR, BRI, CI. BAI and AVI.

[b]Covariates in the multivariable model included age, diabetes, smoking and alcohol history, regular exercise habits, fasting glucose, hemoglobin, total cholesterol, HDL-cholesterol, LDL-cholesterol, eGFR and uric acid for LAP.

[c]Covariates in the multivariable model included age, diabetes, smoking and alcohol history, regular exercise habits, fasting glucose, hemoglobin, total cholesterol, LDL-cholesterol, eGFR and uric acid for VAI.

Supplemental Table S2. Comparison of the association of obesity-related indices with incident hypertension using different logistic regression analysis in female participants

Obesity-related indices	Female (n = 14,567)			Female (n = 14,567)		
	Multivariable#			Multivariable[a,b,c]		
	OR	95% confidence interval	p	OR	95% confidence interval	p
BMI (per 1 kg/m^2)	1.133#	1.115-1.151	< 0.001	1.119[a]	1.101-1.137	< 0.001
WHR (per 0.01)	1.035#	1.027-1.044	< 0.001	1.027[a]	1.018-1.035	< 0.001
WHtR (per 0.01)	1.061#	1.052-1.071	< 0.001	1.053[a]	1.043-1.062	< 0.001
BRI (per 1)	1.197#	1.164-1.232	< 0.001	1.166[a]	1.131-1.201	< 0.001
CI (per 0.1)	1.144#	1.076-1.217	< 0.001	1.095[a]	1.028-1.166	0.005
BAI (per 1)	1.086#	1.071-1.102	< 0.001	1.078[a]	1.062-1.093	< 0.001
AVI (per 1)	1.108#	1.090-1.128	< 0.001	1.090[a]	1.071-1.110	< 0.001
LAP (per 1)	1.008#	1.006-1.010	< 0.001	1.010[b]	1.007-1.013	< 0.001
VAI (per 1)	1.083#	1.053-1.115	< 0.001	1.095[c]	1.062-1.128	< 0.001

Values expressed as odds ratio (OR) and 95% confidence interval. Abbreviations are the same as in Table 1.

#Covariates in the multivariable model included age, smoking and alcohol history, regular exercise habits, hemoglobin, eGFR and uric acid for BMI, WHR, WHtR, BRI, CI. BAI, AVI, LAP and VAI.

[a]Covariates in the multivariable model included age, diabetes, smoking and alcohol history, regular exercise habits, fasting glucose, hemoglobin,

triglyceride, total cholesterol, HDL-cholesterol, LDL-cholesterol, eGFR and uric acid for BMI, WHR, WHtR, BRI, CI. BAI and AVI.

[b]Covariates in the multivariable model included age, diabetes, smoking and alcohol history, regular exercise habits, fasting glucose, hemoglobin, total cholesterol, HDL-cholesterol, LDL-cholesterol, eGFR and uric acid for LAP.

[c]Covariates in the multivariable model included age, diabetes, smoking and alcohol history, regular exercise habits, fasting glucose, hemoglobin, total cholesterol, LDL-cholesterol, eGFR and uric acid for VAI.
